# Nitrite as a pharmacological intervention for the successful treatment of crush syndrome

**DOI:** 10.14814/phy2.13633

**Published:** 2018-03-07

**Authors:** Jun Kobayashi, Isamu Murata

**Affiliations:** ^1^ Division of Pathophysiology Department of Clinical Dietetics and Human Nutrition Faculty of Pharmacy and Pharmaceutical Science Josai University Saitama Japan; ^2^ Division of Drug Safety Management Faculty of Pharmacy and Pharmaceutical Science Josai University Saitama Japan

**Keywords:** Crush syndrome, ischemia/reperfusion injury, nitrite, pre/postconditioning, reactive oxygen species

## Abstract

Crush syndrome is characterized by ischemia/reperfusion injury (IRI). The protective effect of nitrite on experimentally induced IRI has been demonstrated in the heart, kidney, liver, and skeletal muscle. IRI in tissues and systemic organs occurs due to the massive generation of reactive oxygen species and subsequent systemic inflammation. Therefore, ischemic pre and postconditioning are performed in clinical practice. Intravenous administration of nitrite inhibits IRI through nitric oxide‐mediated mechanisms. In this paper, we discuss the utility of nitrite as a pharmacological postconditioning agent in the treatment of crush syndrome.

## Introduction

**Figure 1:**
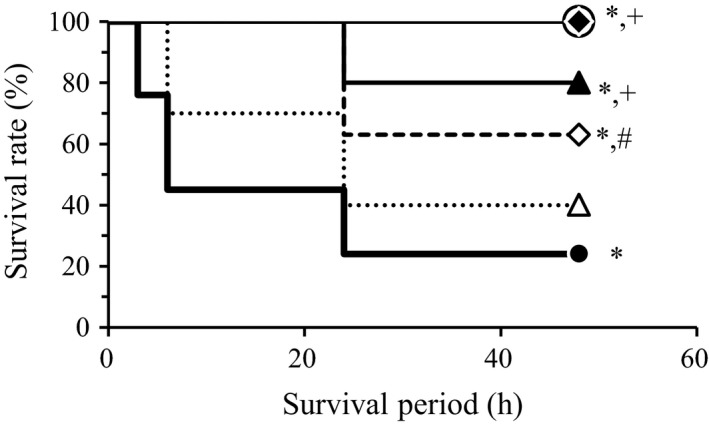
Survival rates associated with different treatments in a rat model of crush syndrome (CS). Data published by Murata et al.(2012, 2013, 2017a,b) are reproduced after modification; sham group, surgically treated rats without CS (○: *n* = 10); CS group, untreated rats with CS (●:*n* = 29); saline group, CS rats treated with saline infusion (30 mL/kg/h for 3 h) for volume expansion following reperfusion (▵:*n* = 10); nitrite i.v. group, CS rats treated with 200 *μ*mol/kg nitrite injection (◇:*n* = 14); bicarbonate with saline group, CS rats treated with bicarbonate admixed saline infusion (30 mL/kg/h for 3 h)(▲: *n* = 10); 200 *μ*mol/L nitrite with saline group, CS rats treated with 200 *μ*mol/kg nitrite in saline infusion (30 mL/kg/h for 3 h) (◆:*n* = 10) * *P* < 0.05 vs. sham, # *P* < 0.05 vs. CS, + *P* < 0.05 vs. saline, by log‐rank test.

Crush syndrome (CS) develops due to the sudden decompression of limb muscles (reperfusion injury) following prolonged compression (ischemia) (Murata et al. 2011). Beneficial effects of nitrite in the treatment of ischemia/reperfusion injury (IRI) in the cardiac muscle have been reported (Bryan et al. 2007; Gonzalez et al. 2008; Carlström et al. 2011); however, its effects in the skeletal muscle are not well‐known. Murata et al. (2012) have demonstrated the therapeutic efficiency of nitrite using animal models of acute CS. They reported that conventional saline infusion and bicarbonate hydration therapies are associated with survival rates of approximately 40 and 80%, respectively, at 48 h after reperfusion, as opposed to 20% survival in untreated CS control animals (Murata et al. 2012, 2017b). However, an intravenous injection of nitrite, immediately before reperfusion, raised the survival rate above 60%. Furthermore, rats treated with continuous nitrite infusion for 3 h, following reperfusion with saline for hypovolemia correction, exhibited 100% survival (Murata et al. 2012, 2013, 2017b) (Fig. 1). They also showed that nitrite levels in the skeletal muscles decreased due to prolonged ischemia. Similar results have been reported for ischemia in the heart and liver tissues in animal models of IRI. Nitrite is an on‐demand nitric oxide (NO) donor, as well as a nitrosating agent serving as a signaling molecule and a gene regulator, especially in hypoxic tissues, by posttranslational protein modification at heme and sulfhydryl sites (Bryan et al. 2005; Kobayashi et al. 2015). Therefore, increasing endogenous and exogenous nitrite levels seems beneficial for the treatment of CS.

## IRI is Responsible for the Development of CS

An important characteristic feature of CS is the systemic inflammatory response following reperfusion to ischemic local skeletal muscles (Murata et al. 2013). Individuals trapped under heavy debris due to building collapse or bombing may have their extremities compressed; such individuals may not experience pain once these body parts have become numb due to lack of blood supply. However, if the crushing pressure is suddenly removed, oxygenated blood rushes into these ischemic tissues (reperfusion), resulting in further damage. This is followed by rhabdomyolysis and the release of proinflammatory mediators to the lungs and kidneys, leading to systemic inflammation. This devastating cascade of events results in life‐threatening conditions, such as circulatory shock, renal failure, and acute respiratory distress syndrome (Sever et al. 2002).

## Ischemic Duration of Skeletal Muscles is Critical for Preventing Mortality Following Reperfusion

Mortality rates associated with CS vary depending on the ischemic duration of skeletal muscles. Ischemic duration directly correlates with the extent of tissue damage. Almost complete survival may be expected if the tissue damage is less; however, ATP depletion and osmotic swelling make the cells more susceptible to cell disruption following reperfusion (Ferdinandy et al. 2007). A prolonged ischemic duration leads to a no‐reflow phenomenon characterized by microvascular occlusion in the infarct core. The only treatment to ensure survival is to amputate the necrotic limbs. The identification of ischemic duration is critical to decide the treatment approach. Murata et al. (2011) reported that 5 h of tourniquet‐induced skeletal muscle ischemia in rats resulted in 100% acute phase mortality within 24 h after reperfusion; however, mortality dropped to 0 and 10% with ischemic durations of 4 and 6 h, respectively. No systemic release of inflammatory mediators was observed with crush periods less than 4 h (less tissue damage) and greater than 6 h (no‐reflow phenomenon). The microvasculature is completely occluded following ischemic durations of 6 h or more (Blaisdell 2002). Although the critical ischemic period differs with the organ, animal species, and experimental device used for inducing ischemia, a crush period of 5 h in rats effectively simulates lethal CS with severe rhabdomyolysis and subsequent acute circulatory, respiratory, and renal failures (Murata et al. 2011).

## Nitrite is Depleted in Ischemic Tissues

Tissue nitrite levels decrease after persistent ischemia in the liver, heart, and skeletal muscles (McNulty et al. 2008; Raat et al. 2009a), indicating nitrite depletion during ischemia. Tissue nitrite serves as an NO reservoir and NO donor under hypoxic and acidic conditions in the heart and skeletal muscles. The released NO and NO^+^ then drive various signaling pathways through the activation of soluble guanylate cyclase and/or cGMP‐independent transnitrosation (Tiravanti et al. 2004), and plays an important role in protecting the cells and tissues during ischemia. It induces hypoxic vasodilation, reduces oxygen consumption (by inhibiting mitochondrial complexes I, IV, and V), opens K_ATP_ channels, prevents cytochrome c release, and blocks the opening of mitochondrial permeability transition pore (mPTP). Consistent with these effects, orally, intraperitoneally, or intravenously administered nitrite attenuates IRI‐induced damage to the heart, brain, kidney, and liver. Mice fed a diet deficient in nitrate and nitrite for 7 days exhibit diminished plasma and heart levels of nitrite and show severe myocardial infarction following experimentally induced IRI. However, nitrite supplementation attenuated myocardial infarction in a dose‐dependent manner (Bryan et al. 2007). This suggests that endogenous nitrite confers tolerance to ischemia. Therefore, prophylactic nitrite administration may be effective in protecting the tissues from IRI.

**Figure 2:**
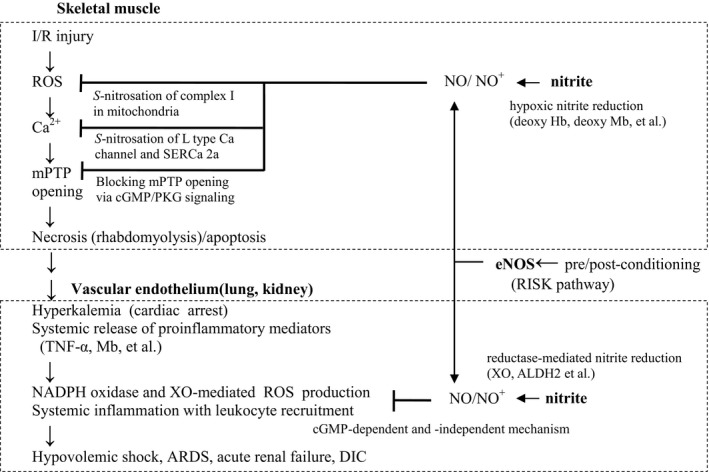
Schematic representation of IRI and RISK pathways linked to NO‐mediated cytoprotection. Pre and postconditioning phosphorylate PI3K‐Akt, and MEK‐1/2‐ERK‐1/2 cascades, which are also associated with phosphorylation of eNOS (RISK pathway) (Liem et al. 2007). Pharmacological postconditioning with nitrite shares cGMP‐dependent and independent (nitrosation and nitrosylation) pathways with the RISK pathway (Liem et al. 2007). The major mediators of post‐IRI are ROS production, dysregulated intracellular Ca^2+^ overload, and mPTP opening. Nitrite inhibits ROS generation by nitrosation of complex I in the mitochondrial respiratory chain. Nitrite inhibits cytosolic Ca^2+^ overload by nitrosation of L‐type Ca^2+^ channels (inhibiting Ca^2+^ release) and SERCA2a (activating Ca^2+^ uptake) (Sun et al. 2007). Nitrite also inhibits mPTP opening by phosphorylating serine/threonine residues of mitochondrial proteins by activating cGMP/PKG signaling (Kim et al. 2004). Then, the IRI in skeletal muscle enters the second stage, where systemic inflammation occurs in the vascular endothelium of vital organs including kidney and lung. Although nitrite remarkably reduces rhabdomyolysis and systemic release of proinflammatory mediators by decreasing cell vulnerability to reperfusion, abnormal interactions of leukocytes with systemic vascular endothelium are inhibited by nitrite‐derived NO/N_2_O_3_, preventing lethal complications (hypovolemic shock, ARDS, acute renal failure, and DIC) to secondary systemic inflammation (Murata et al. 2017b). RISK pathway, reperfusion injury salvage kinase pathway; IRI, ischemia/reperfusion injury; PI3K, phosphoinositide 3‐kinase; MEK‐1/2, MAPK (Mitogen‐Activated Protein Kinase)/ERK (Extracellular signal‐Regulated Kinases) kinase‐1/2; ROS, reactive oxygen species; mPTP, mitochondrial permeability transition pore; NO, nitric oxide; eNOS, endothelial NO synthase; ARDS, acute respiratory distress syndrome; DIC, disseminated intravascular coagulation; XO, xanthine oxidase; TNF, tumor necrosis factor; ALDH, aldehyde dehydrogenase; Hb, hemoglobin; Mb, myoglobin; SERCA, sarco/endoplasmic reticulum Ca²⁺‐ATPase.

Ischemic preconditioning involves exposing the tissues to several brief intervals of sublethal ischemia followed by reperfusion to induce low levels of reactive oxygen species (ROS). This activates the reperfusion injury salvage kinase (RISK) pathway, which involves Akt/endothelial NO synthase (eNOS) activation and subsequent NO‐mediated cytoprotective signaling (Kim et al. 2004; Ferdinandy et al. 2007) (Fig. 2). However, due to the unpredictable nature of the onset of crush injury, pharmacological preconditioning with NO is impractical. Nevertheless, it may be useful as an onsite postconditioning agent immediately before reperfusion.

## Nitrite as a Pharmacological Postconditioning Agent for the Acute Phase Treatment of IRI

Postconditioning involves tissue exposure to brief alternating periods of ischemia and reflow, applied at the onset of reperfusion, following sustained ischemia (Tsang et al. 2004). Postconditioning was initially thought to attenuate IRI by gradually reintroducing oxygen to ischemic tissues or diluting toxic metabolites at the ischemic site; however, it was found to activate the RISK pathway involving survival PI3K/Akt pathways leading to NO‐mediated signaling (Hausenloy et al. 2005) (Fig. 2). This has been verified by pharmacological inhibition studies of RISK pathway components (Tsang et al. 2004) and the measurement of the levels and enzymatic activities of eNOS and inducible NOS (Brunner et al. 2003; Elrod et al. 2006; West et al. 2008). Manipulation of these enzymes by knockout or transgenic overexpression studies in IRI animal models further highlights the role of postconditioning in CS treatment (Ozaki et al. 2002).

Sudden oxygen rush to anoxic tissues following reperfusion generates a large amount of ROS, particularly at mitochondrial complex I (the entry point for electrons from NADH into the respiratory chain), followed by Ca^2+^ overload, mPTP opening, and cytochrome c release, leading to cell necrosis/apoptosis. Therefore, ROS generation after reperfusion could be responsible for ischemic tissue damage and subsequent systemic release of proinflammatory mediators, which react with the vascular endothelium and cause multiple organ failures (Murata et al. 2011). Nitrite protects the hypoxic tissues from reperfusion injury by limiting ROS generation via posttranslational S‐nitrosylation of complex I (at Cys39 on the ND3 subunit), which becomes susceptible to this modification only during ischemia (Chouchani et al. 2013). In addition, the cytoprotective effects of nitrite may be mediated by S‐nitrosylation and S‐glutathionylation of redox‐sensitive cysteine residues of Ca^2+^ handling channels and receptors (L‐type Ca^2+^ channel, ryanodine receptor type 1, and sarcoendoplasmic reticulum Ca^2+^ transport ATPase) to prevent ROS‐induced oxidation and abnormal intracellular Ca^2+^ handling (Aracena‐Parks et al. 2006; Sun et al. 2007; Sun and Murphy 2010). Although detailed mechanisms are not fully known, these effects attenuate the increase in cytosolic Ca^2+^ during ischemia and Ca^2+^ overload during reperfusion.

An intravenous infusion of nitrite confers greater protection against IRI than a bolus intravenous injection in CS rats (Fig. 2). Systemically infused nitrite is catalyzed to NO by aldehyde dehydrogenase 2 (Sonoda et al. 2014), and reacts with deoxyhemoglobin to form NO‐hemoglobin (Lundberg et al. 2008). Due to the inflammatory surge during ischemia, leukocytes abnormally interact with the vascular endothelium and lead to secondary ROS generation induced by leukocyte NADPH oxidase and vascular xanthine oxidase (Raat et al. 2009b). NO and its metabolites prevent this interaction.

Intravenous injection of dexamethasone (Murata et al. 2013) and astragaloside IV, the primary pure saponin isolated from *Astragalus membranaceus*, have potent anti‐inflammatory and antioxidant actions, and are associated with 100% survival rates in CS rat models (Murata et al. 2017a). Similar to nitrite, they enhance eNOS phosphorylation, inhibit ROS production, and aid pre and postconditioning in IRI.

## Safety Concerns About Nitrite Infusion

Murata et al. (2012) demonstrated that 200 *μ*mol/kg of intravenously administered nitrite has no impact on mean arterial pressure and methemoglobin levels. In addition, rats treated with continuous saline infusion along with 200 *μ*mol/kg of nitrite for 3 h after reperfusion exhibited 100% survival without any hypotensive effect or methemoglobinemia (Murata et al. 2017b). On the other hand, Pluta et al. (2011) reported the safety and feasibility of intravenous sodium nitrite infusion in healthy volunteers. They showed that a dose of 20 *μ*mol/kg/3 h leads to nitrite‐limiting toxicity, including a transient asymptomatic decrease (>15 mmHg) in mean arterial pressures and/or an asymptomatic increase (>5%) in methemoglobin levels in humans. Although lower doses of nitrite need to be evaluated for the treatment of CS, these results specify the effective lower limit of nitrite dose in the clinical treatment of CS.

## Conclusion

In addition to conventional infusion therapy for extracellular fluid volume expansion and acid‐base control, nitrite infusion therapy is a promising pharmacological intervention for the treatment of CS through a protective mechanism involving NO‐mediated signaling. Therefore, we propose that treatment with low‐cost nitrite infusion should be clinically implemented to prevent IRI and as a potential therapeutic agent for CS.

## Conflict of Interest

None declared.
